# Anti-PD-1/PD-L1 antibodies versus docetaxel in patients with previously treated non-small-cell lung cancer

**DOI:** 10.18632/oncotarget.23584

**Published:** 2017-12-21

**Authors:** Qi Jiang, Mixue Xie, Mengye He, Feifei Yan, Xiaochen Zhang, Sufen Yu

**Affiliations:** ^1^ Department of Medical Oncology, The First Affiliated Hospital, College of Medicine, Zhejiang University, Hangzhou, Zhejiang Province, 310003, China; ^2^ Senior Department of Haematology, The First Affiliated Hospital, College of Medicine, Zhejiang University, Hangzhou, Zhejiang Province, 310003, China

**Keywords:** PD-1, PD-L1, immune checkpoint inhibitor, non–small cell lung cancer, meta-analysis

## Abstract

Anti-PD-1/PD-L1 antibodies have been proved one of the most promising treatments against non-small cell lung cancer (NSCLC); however, whether anti-PD-1/PD-L1 antibodies can provide added benefits for pretreated patients with advanced NSCLC and which patients are most likely to benefit from anti-PD-1/PD-L1 therapy remain controversial. This meta-analysis evaluated the efficacy and safety between anti-PD-1/PD-L1 antibodies and docetaxel in previously treated, advanced NSCLC. PubMed, EMBASE and Cochrane library databases were systematically searched for eligible studies. Five studies with a total of 3,025 patients were included. Our results showed that, for all patients, anti-PD-1/PD-L1 therapy prolonged overall survival (OS) (hazard ratio [HR] = 0.69; 95% CI, 0.63–0.75) and progression-free survival (PFS) (HR = 0.87; 95% CI, 0.80–0.94). For patients with PD-L1 expression ≥1%, anti-PD-1/PD-L1 therapy had higher objective response rates. In subgroup analysis according to the tumor PD-L1 expression level, anti-PD-1/PD-L1 therapy was associated with longer OS and PFS in patients with high PD-L1 expression (≥1%, ≥5%, ≥10% and ≥50%), but not in those with low expressions. In subgroup analysis of patients’ characteristics, anti-PD-1/PD-L1 antibodies showed OS benefits across most prespecified subgroups, except for patients with *EGFR* mutation-positive and never smokers. For patients with *EGFR* mutation, anti-PD-1/PD-L1 therapy was an unfavorable factor of PFS. The grade 3 or 4 adverse events rates of anti-PD-1/PD-L1 treatment were significantly lower than that of docetaxel. Our results suggest that anti-PD-1/PD-L1 therapy significantly improves survival compared with docetaxel in patients with previously treated, PD-L1-positive, advanced NSCLC, and has a distinct safety profile from chemotherapy.

## INTRODUCTION

One of the commonest cancers and the main cause of cancer-related death worldwide is lung cancer [1]. Non–small cell lung cancer (NSCLC) is diagnosed in about 85% of patients with lung cancer; about 80% cases are at an advanced stage at the time of diagnosis [2–3]. Traditional therapies, including molecular targeted therapy, have demonstrated an increasing number of limitations because of drug resistance. Second-line docetaxel treatment confers modest benefit to patients with worsened disease after initial treatment. Furthermore, the adverse events following docetaxel treatment are poorly tolerated [4–5].

Following greater understanding of the multiple tumor immune escape mechanisms in recent years, programmed cell death 1/programmed cell death 1 ligand 1 (PD-1/PD-L1) pathway inhibition, followed by immune system tumor killing effect reactivation has become a new strategy for treating cancer [6–7]. A series of phase II/III randomized trials on the efficacy and toxicities of anti-PD-1/PD-L1 antibodies as compared with docetaxel for advanced NSCLC have been conducted [8–12]. The trials demonstrated that PD-L1/PD-1 pathway inhibition has encouraging results for survival for all NSCLC subtypes. However, accurate identification of patients suitable for this immunotherapeutic strategy via therapeutic predictive biomarkers is inconsistent and inconclusive [13]. In addition, largely owing to the relatively small sample sizes of the individual studies, the results have been controversial. Therefore, we performed this meta-analysis, systematically combining data from published clinical trials, to evaluate the efficacy and safety of anti-PD-1/PD-L1 antibodies in previously treated advanced NSCLC as compared to docetaxel.

## RESULTS

### Study selection

**Figure 1:**
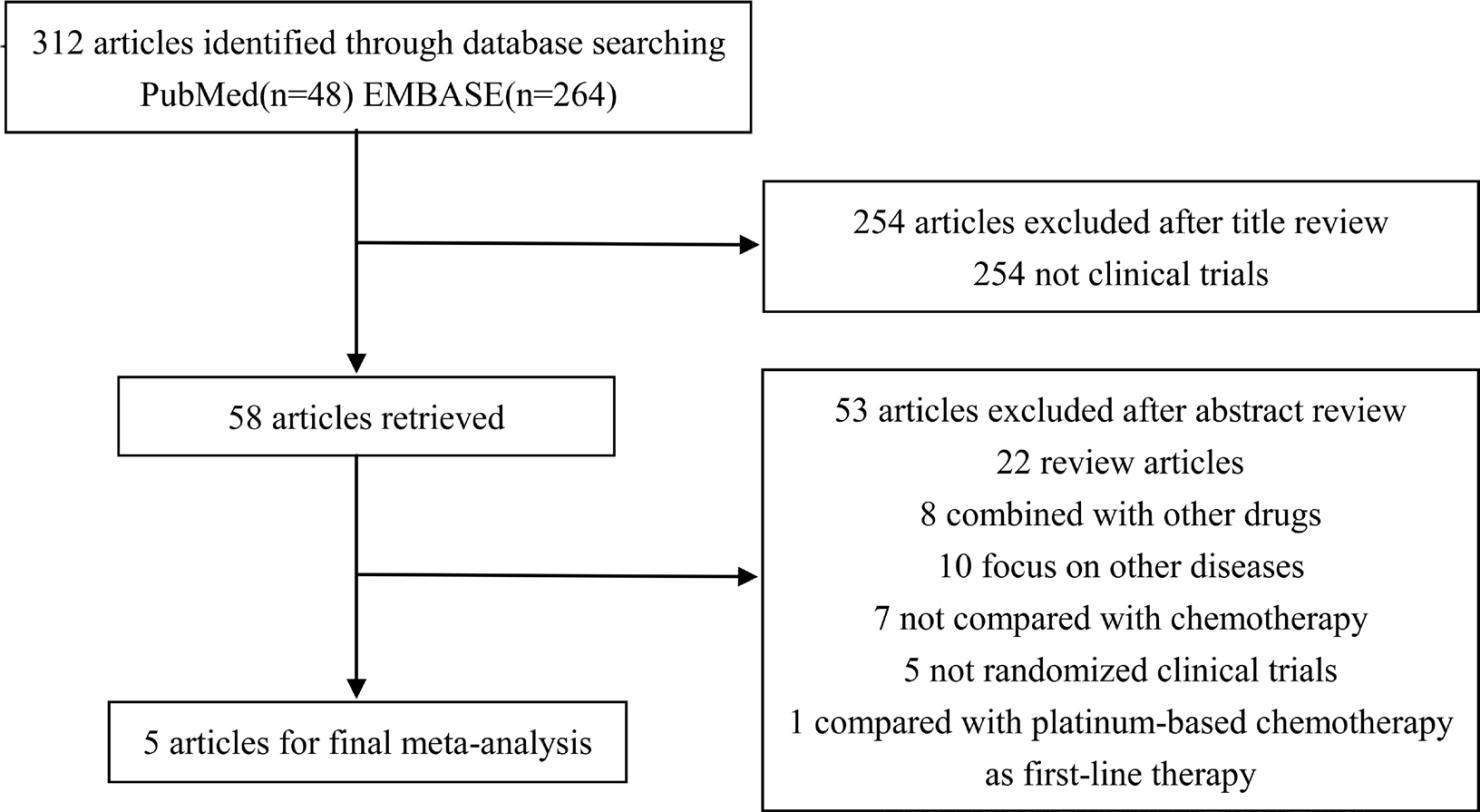
Flow diagram of the study selection process

**Figure 2:**
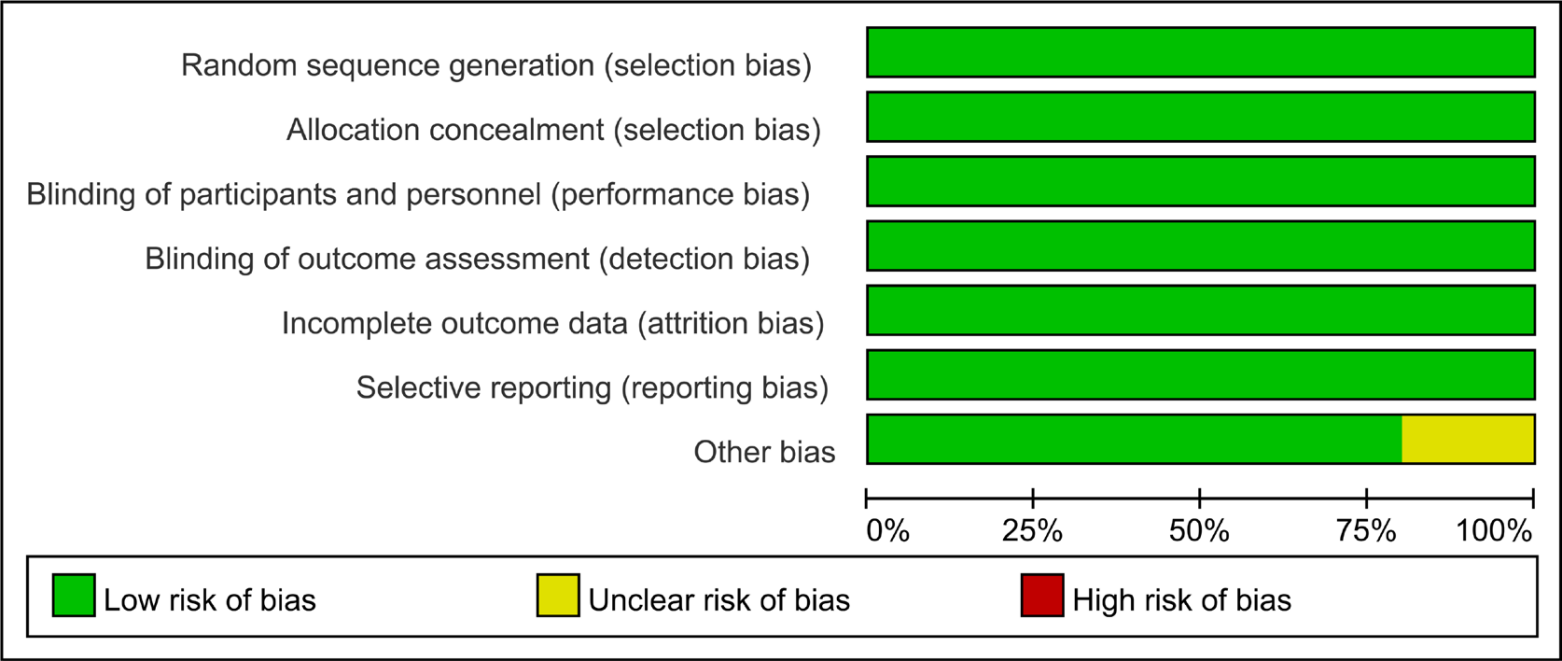
Risk of bias percentile chart

The search strategy identified 312 records that were screened for inclusion. Title review determined that 254 studies were not clinical trials, and the studies were excluded. Abstract review excluded 53 studies which did not meet the selection criteria. In total, five trials performed between the year 2012 and 2015, which included 3,025 patients, met the inclusion criteria. (Figure 1) All the included trials were of high quality with low bias of selection, performance, detection, attrition and reporting (Figure 2).

### Study characteristics

Table 1 lists the characteristics of the five trials. Of the five publications included in the meta-analysis, CheckMate 057 [8], CheckMate 017 [9] and KEYNOTE-010 [11] examined the effect of the anti-PD-1 antibodies (Nivolumab and Pembrolizumab), while POPLAR [10] and OAK [12] evaluated the anti-PD-L1 antibody (Atezolizumab). The median participant age was 61–64 years; in the studies that reported on participant sex, 52–82% of participants were men. The Eastern Cooperative Oncology Group (ECOG) performance status scores of almost all patients in the five trials were between 0 and 1. All trials used the Response Evaluation Criteria in Solid Tumors (RECIST; version 1.1) and the National Cancer Institute Common Terminology Criteria for Adverse Events (version 4.0) for assessing tumor response and treatment-related adverse events, respectively. Subgroup analyses in these trials explored the correlations between immunotherapy efficacy and the patient features age; sex; region; race; line of therapy; NSCLC types; smoking status; ECOG score; PD-L1 expression; and *EGFR*, *KRAS*, or *ALK* gene mutation status.

**Table 1:** Characteristics of each study

Author and Study	Year	Research Period	Phase	Prior Therapy	NSCLC Histology	Drug	Usage and Dosage (Median Doses or Time)	No. of Patients	Age (Years), Median (Range)	Male (%)	Tobacco Use History(%)
**Borghaei****[8]** **CheckMate 057**	2015	2012.12–2013.12	3	1	Non-squamous	Nivolumab (PD-1 antibody)	3 mg/kg, intravenously, every 2 weeks (6 doses)	292	61 (37–84)	151 (52%)	231 (79%)
						Docetaxel	75 mg/m^2^, intravenously, every 3 weeks (4 doses)	290	64 (21–85)	168 (58%)	227 (78%)
**Brahmer****[9]** **CheckMate 017**	2015	2012.10–2013.12	3	1	Squamous	Nivolumab (PD-1 antibody)	3 mg/kg, intravenously, every 2 weeks (8 doses)	135	62 (39–85)	111 (82%)	121 (90%)
						Docetaxel	75 mg/m^2^, intravenously, every 3 weeks (3 doses)	137	64 (42–84)	97 (71%)	129 (94%)
**Fehrenbacher****[10]** **POPLAR**	2016	2013.08–2014.03	2	1 or 2	All	Atezolizumab (PD-L1 antibody)	1200 mg, intravenously, every 3 weeks (3.7 months)	144	62 (42–82)	93 (65%)	117 (81%)
						Docetaxel	75 mg/m^2^, intravenously, every 3 weeks (2.1 months)	143	62 (36–84)	76 (53%)	114 (80%)
**Herbst****[11]** **KEYNOTE-010**	2016	2013.08–2015.02	2/3	≥1	Squamous and adenocarcinoma	Pembrolizumab (PD-1 antibody)	2 mg/kg, intravenously, every 3 weeks (3.5 months)	345	63 (56–69)	212 (62%)	279 (81%)
						Pembrolizumab (PD-1 antibody)	10 mg/kg, intravenously, every 3 weeks (3.5 months)	346	63 (56–69)	213 (62%)	285 (82%)
						Docetaxel	75 mg/m^2^, intravenously, every 3 weeks (2.0 months)	343	62 (56–69)	209 (61%)	269 (78%)
**Rittmeyer****[12]** **OAK**	2017	2014.03–2015.04	3	1 or 2	All	Atezolizumab (PD-L1 antibody)	1200 mg, intravenously, every 3 weeks (3.4 months)	425	63 (33–82)	261 (61%)	341 (80%)
							75 mg/m^2^, intravenously, every 3 weeks (2.1 months)	425	64 (34–85)	259 (61%)	353 (83%)

Abbreviations: 1 = one line of therapy; 2 = two lines of therapy; ≥1 = more than one line of therapy; NR = not reported.

### Publication bias

**Figure 3:**
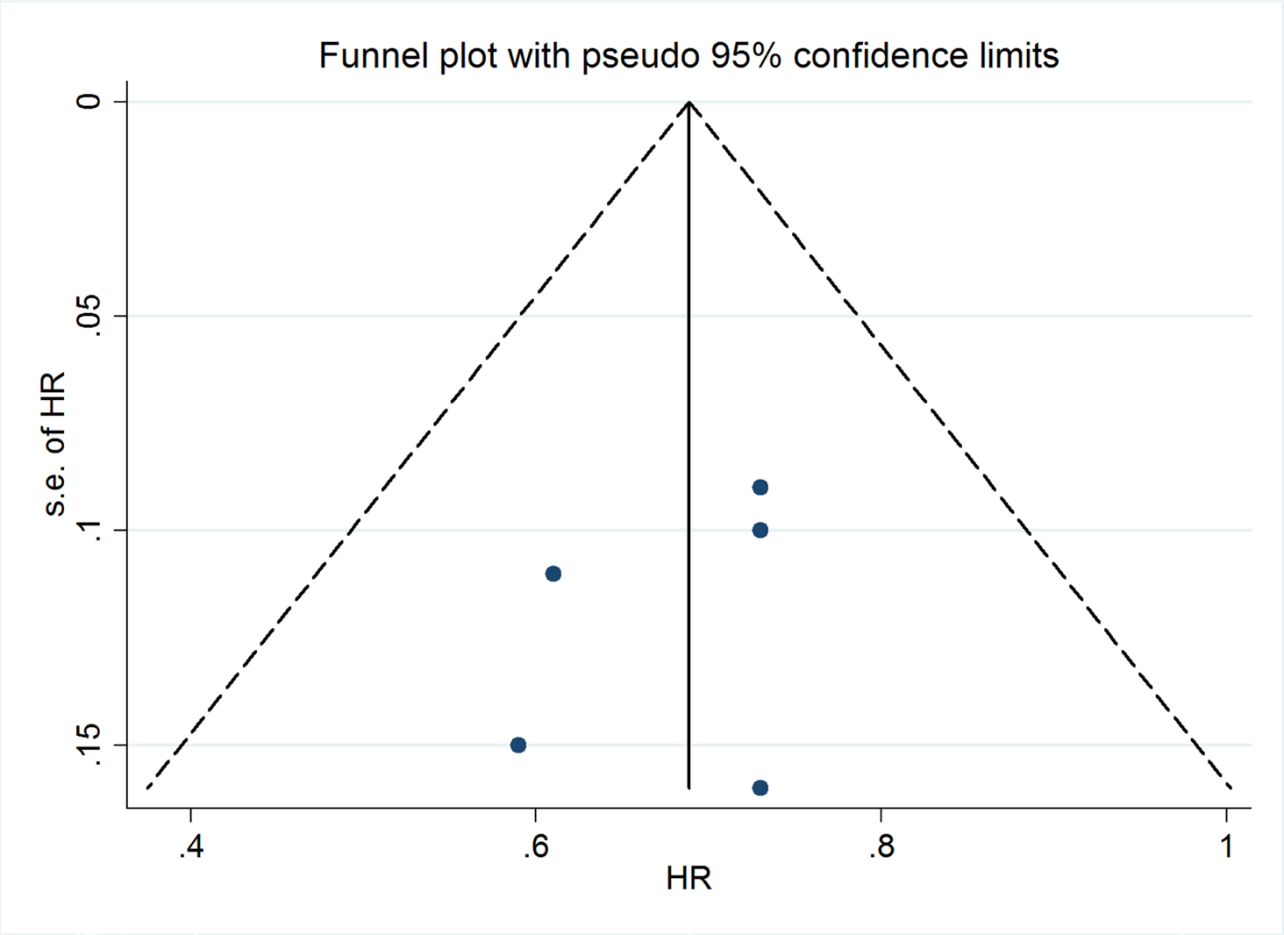
Deek’s funnel plot of included trials

The Deek’s funnel plot and Begg or Egger test demonstrated that there was no evidence of publication bias across the included studies regarding OS (Figure 3; Begg test, *p* = 0.327; Egger test, *p* = 0.500).

### Efficacy of anti-PD-1/PD-L1 antibodies versus docetaxel

**Figure 4:**
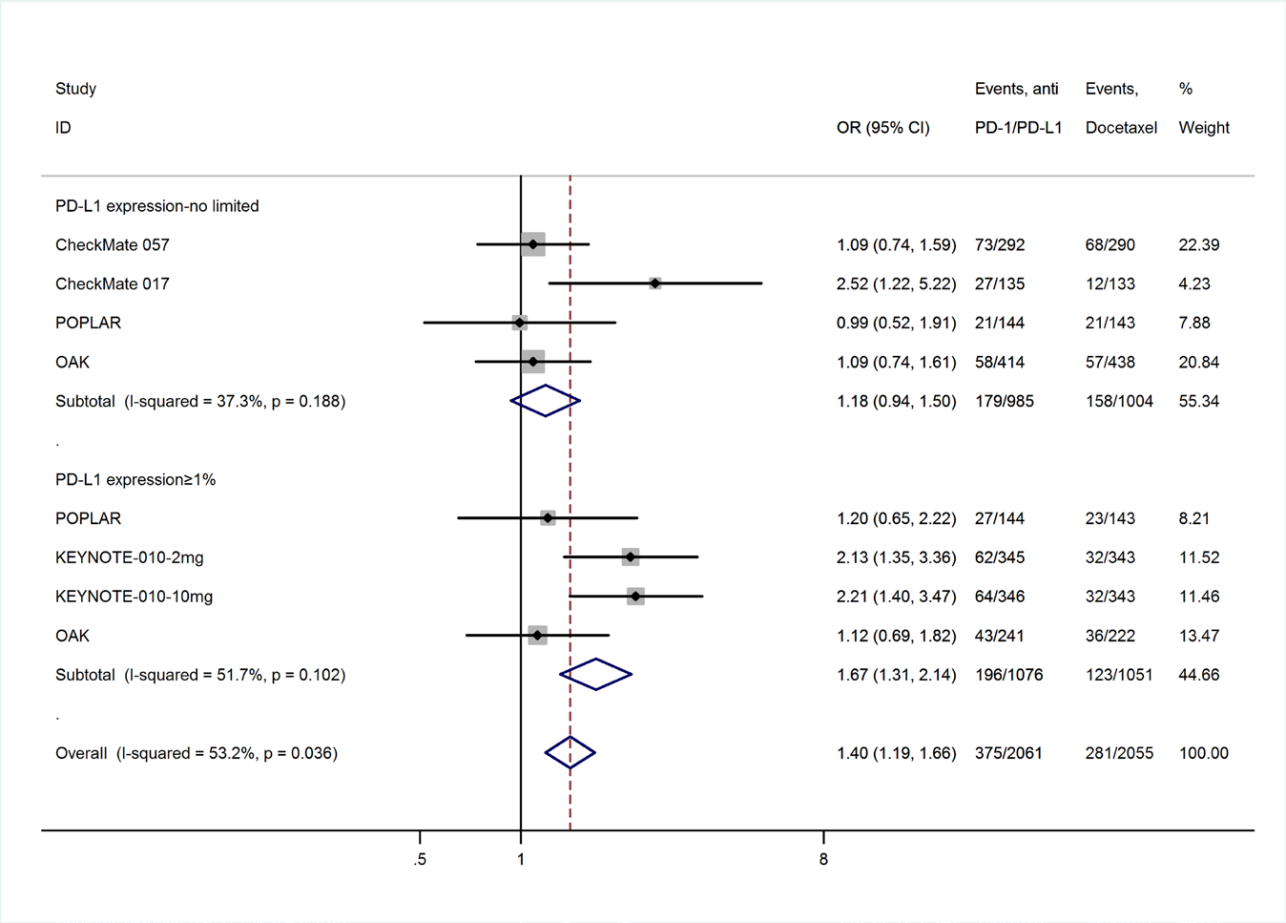
Meta-analysis of OR rates in subgroups of patients with PD-L1 expression ≥1% and no limited

The efficacy endpoint was the objective response (OR) rate, which was reported as complete response (CR) and partial response (PR) rates according to RECIST 1.1. Table 2 lists the response outcomes of the anti-PD-1/PD-L1 antibodies and docetaxel. The heterogeneity test of OR event rates of 3,025 patients from the five studies revealed a Cochran *Q*-test *p*-value of 0.000 and *I*^2^ of 81.5%, indicating high heterogeneity. The sensitivity analysis and meta-regression analysis demonstrated that PD- L1 expression level of included patients in KEYNOTE-010 study (all were >1%) was the main reason of the heterogeneity (Supplementary Table 1). Therefore, the OR event rates were calculated in subgroups of PD-L1 expression level to reduce the heterogeneity between studies. In patients with PD-L1 expression ≥1%, the overall OR rates of patients treated with anti-PD-1/PD-L1 antibodies (18%, 95% confidence interval [95% CI], 16–21%; Supplementary Figure 1) were significantly higher than that of docetaxel-treated patients (14%, 95% CI, 8–19%; Supplementary Figure 1), with an odds ratio of 1.67 (95% CI, 1.31–2.14; *p* = 0.010; Figure 4); while in patients with no or limited PD-L1 expression, there is no difference in OR rates between these 2 drugs, with an odds ratio of 1.18 (95% CI, 0.94–1.50; *p* > 0.05; Figure 4; Supplementary Figure 2).

**Table 2:** Response rates and survival outcomes for anti-PD-1/PD-L1 antibody and docetaxel treatment

Author and Study	Drug	ORNo. (%)	SDNo. (%)	PDNo. (%)	Median OSmonths (95% CI)	1-year OS Rate (95% CI)	Median PFSmonths (95% CI)	1-year PFS Rate (95% CI)
**Borghaei** **[8]****CheckMate 057**	Nivolumab (PD-1 antibody)	73 (25)	103 (35)	111 (38)	12.2 (9.7–15.0)	51% (45%-56%)	2.3 (2.2–3.3)	19% (14%-23%)
	Docetaxel	68 (23	96 (33)	116 (40)	9.4 (8.1–10.7)	39% (33%-45%)	4.2 (3.5–4.9)	8% (5%-12%)
**Brahmer** **[9]****CheckMate 017**	Nivolumab (PD-1 antibody)	27 (20)	3 (29)	56 (41)	9.2 (7.3–13.3)	42% (34%-50%)	3.5 (2.1–4.9)	21% (14%-28%)
	Docetaxel	12 (9)	47 (34)	48 (35)	6.0 (5.1–7.3)	24% (17%-31%)	2.8 (2.1–3.5)	6% (3%-12%)
**Fehrenbacher** **[10]****POPLAR**	Atezolizumab (PD-L1 antibody)	21 (15)	NR	NR	12.6 (9.7–16.4)	51% (NR)	2.7 (2.0–4.1)	17% (NR)
	Docetaxel	21 (15)	NR	NR	9.7 (8.6–12)	41% (NR)	3.0 (2.8–4.1)	12% (NR)
**Herbst** **[11]****KEYNOTE-010**	Pembrolizumab (2 mg/kg) (PD-1 antibody)	62 (18)	NR	NR	10.4 (9.4–11.9)	43.2% (NR)	3.9 (3.1–4.1)	17% (NR)
	Pembrolizumab (10 mg/kg) (PD-1 antibody)	64 (19)	NR	NR	12.7 (10.0–17.3)	52.3% (NR)	4.0 (2.7–4.3)	22% (NR)
	Docetaxel	32 (9)	NR	NR	8.5 (7.5–9.8)	34.6% (NR)	4.0 (3.1–4.2)	8% (NR)
**Rittmeyer** **[12]****OAK**	Atezolizumab (PD-L1 antibody)	58 (14)	159 (35)	187 (44)	13.8 (11.8–15.7)	55% (NR)	2.8 (2.6–3.0)	21% (NR)
	Docetaxel	57 (13)	177 (42)	117 (28)	9.6 (8.6–11.2)	41% (NR)	4.0 (3.3–4.2)	13% (NR)

Abbreviations: OR = objective response; SD = stable disease; PD = progression of disease; NR = not reported.

### Survival advantage of anti-PD-1/PD-L1 antibodies compared with docetaxel

**Figure 5:**
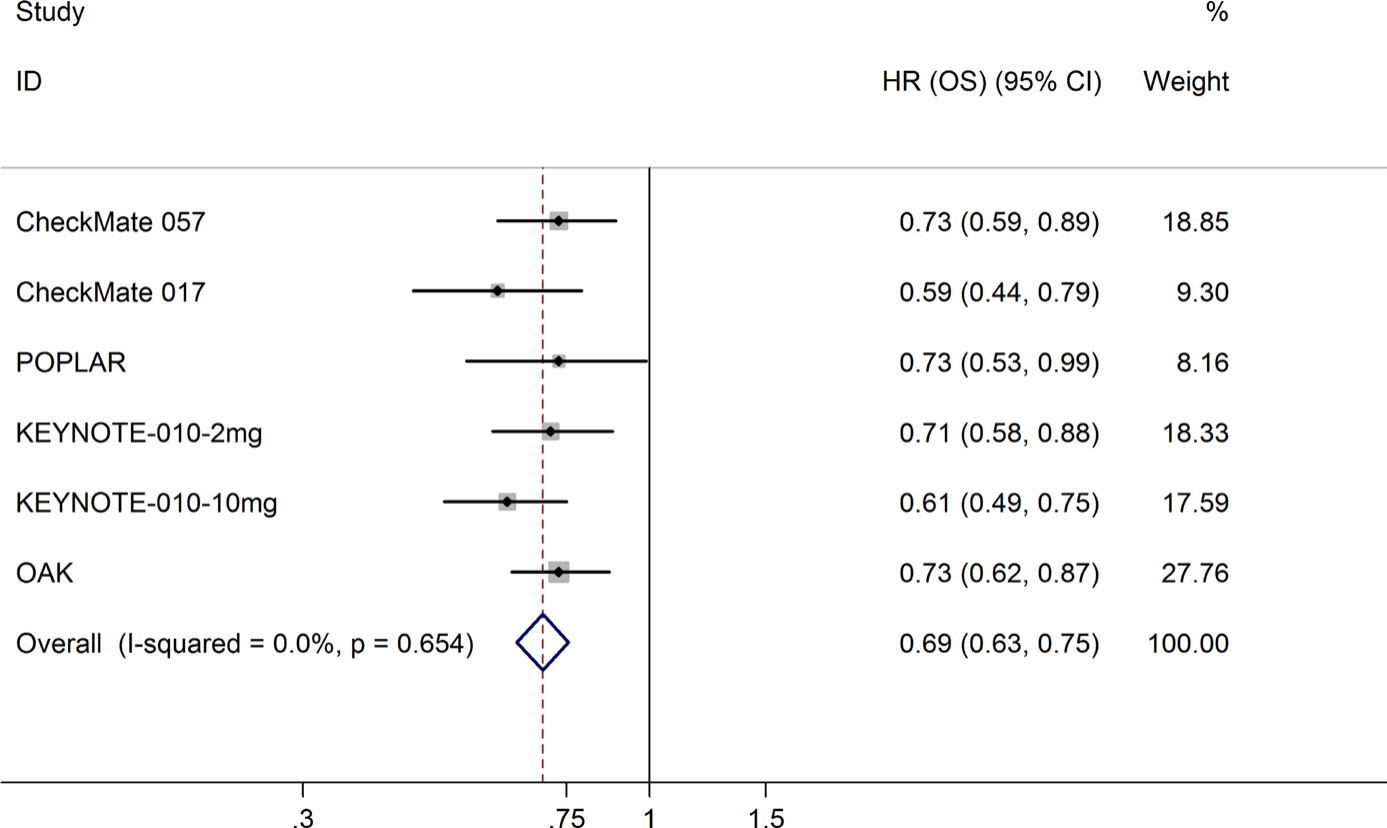
Meta-analysis of OS

**Figure 6:**
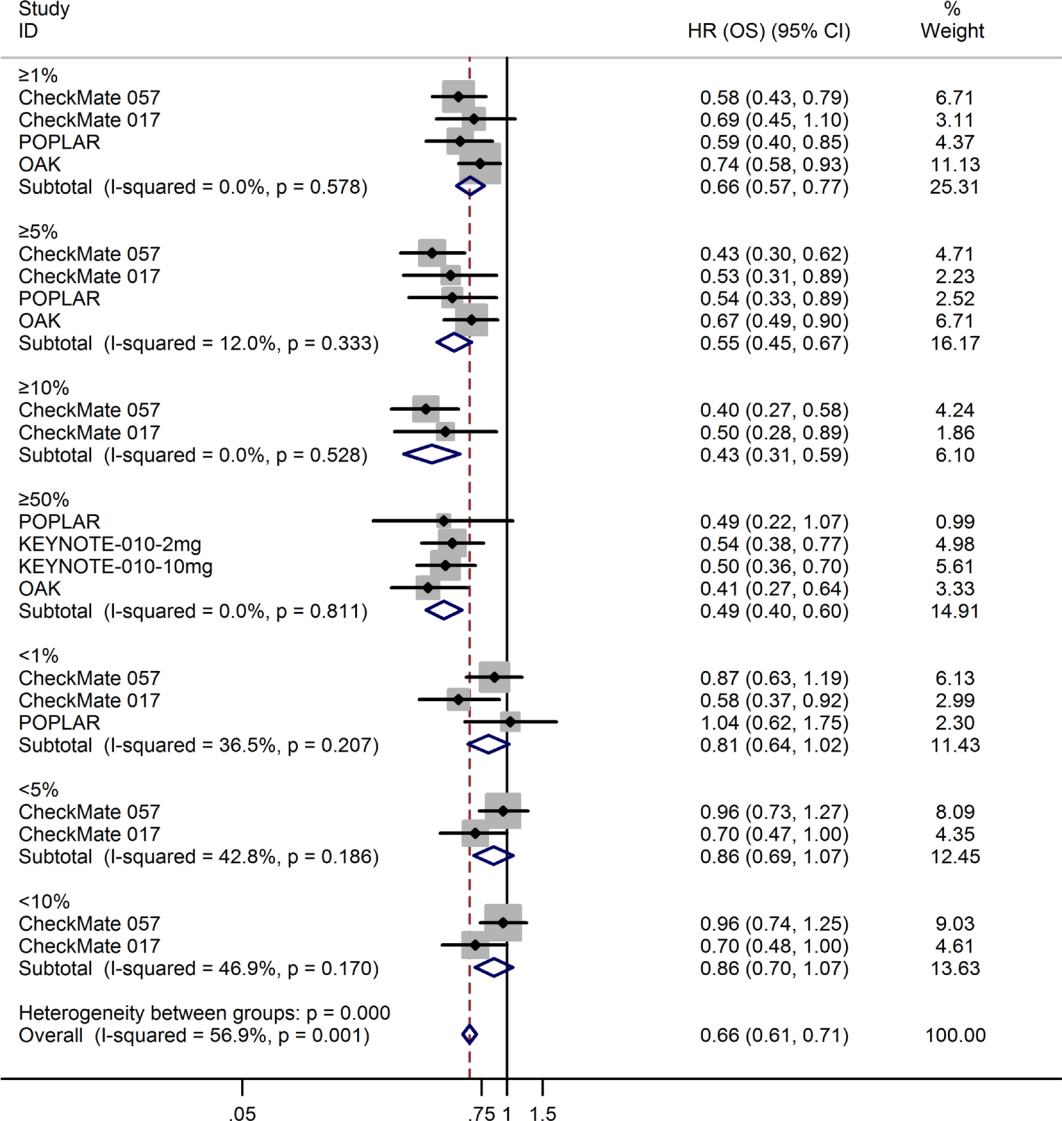
Meta-analysis of OS in patients with 1%, 5%, 10%, and 50% PD-L1 expression

**Figure 7:**
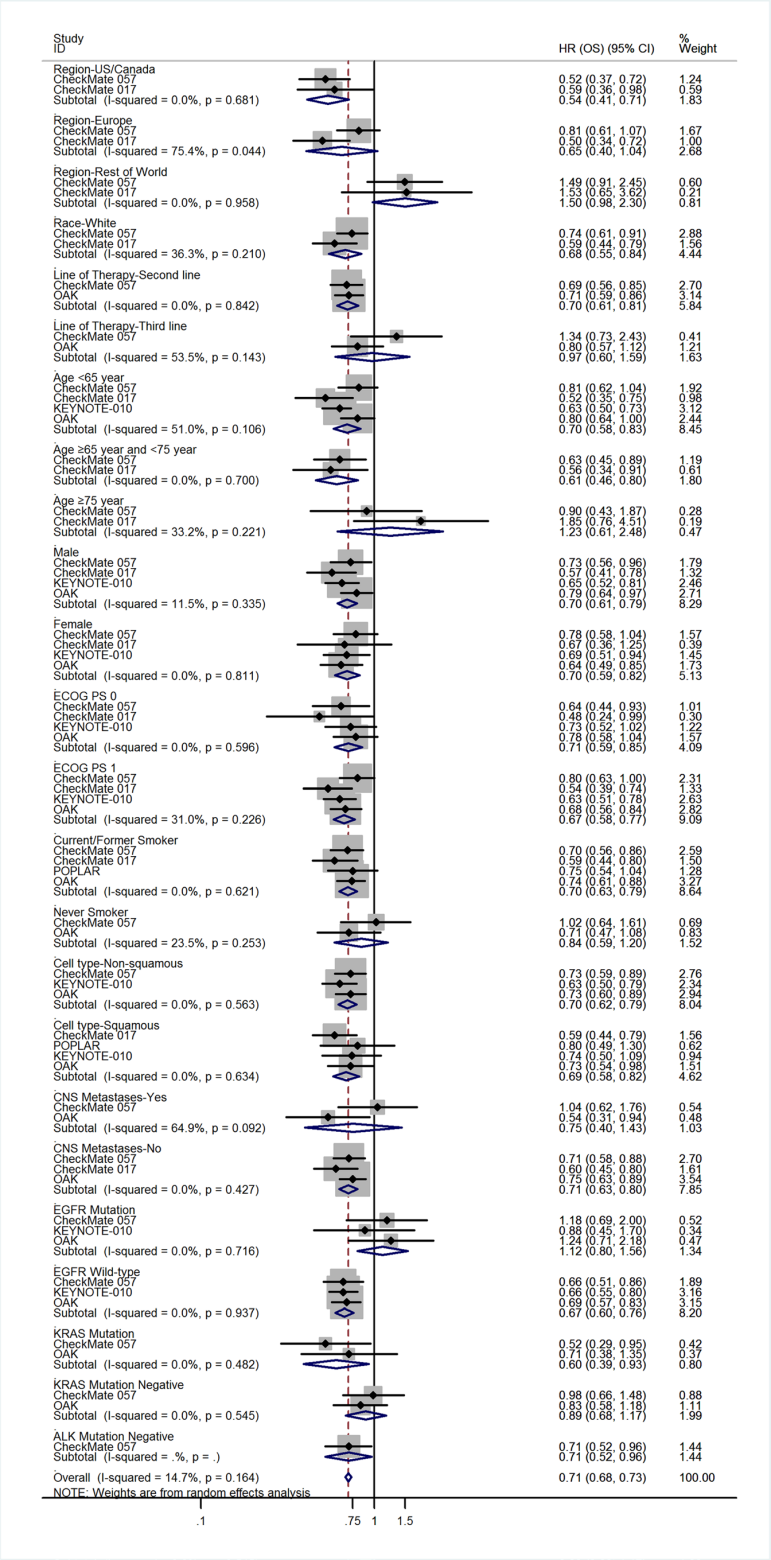
Subgroup analyses of the associations between OS and patient features

All five trials reported the hazard ratios (HRs) for overall survival (OS) and progression-free survival (PFS). Compared with docetaxel, anti-PD-1/PD-L1 antibodies significantly prolonged OS (HR = 0.69; 95% CI, 0.63–0.75; *p* = 0.000; fixed-effects model; Figure 5) and PFS (HR = 0.87; 95% CI, 0.80–0.94; *p* = 0.000; fixed-effects model; Supplementary Figure 3). Table 2 lists other survival data, including median OS, median PFS, 1-year OS rate, and 1-year PFS rate. Three trials [8–9, 11] detected PD-L1 protein expression on tumor cells (TC); the POPLAR study [10] and OAK study [12] detected PD-L1 protein expression on TC and tumor-infiltrating immune cells (IC). For uniform classification of PD-L1 expression levels, TC0 and IC0 (TC < 1% and IC < 1%), TC1/2/3 or IC1/2/3 (TC > 1% or IC > 1%), TC2/3 or IC2/3 (TC > 5% or IC > 5%), and TC3 or IC3 (TC > 50% or IC > 50%) were considered to approximate TC PD-L1 expression levels of <1%, >1%, >5%, and >50%, respectively. In the subgroup analysis of PD-L1 expression level, treatment with anti-PD-1/PD-L1 antibodies favorably influenced both OS (fixed-effects model; Figure 6) and PFS (random-effects model; Supplementary Figure 4) compared with docetaxel in patients with high PD-L1 expression, whereas no advantage was shown for anti-PD-1/PD-L1 antibodies in patients with low PD-L1 expression. The results were similar irrespective of whether PD-L1 expression was categorized as 1%, 5%, 10%, or 50%. The HRs in the analyses of OS favored anti-PD-1/PD-L1 antibodies in the following subgroups: patients in the US/Canada, patients who were white, patients receiving second-line therapy, age <75 years, both sexes, ECOG score of 0–1, history of tobacco use, squamous or adenocarcinoma NSCLC, no central nervous system (CNS) metastases, *EGFR* mutation–negative status, *KRAS* mutation–positive status, and *ALK* mutation–negative status (Figure 7). PFS benefit of immunotherapy was consistent across the following subgroups: patients in the US/Canada, age < 65 years, male patients, ECOG score of 1, history of tobacco use, squamous NSCLC, and *EGFR* mutation–negative status. Subgroup analysis of patients with *EGFR* mutation revealed that docetaxel prolonged PFS when compared with anti-PD-1/PD-L1 antibodies (Supplementary Figure 5).

### Treatment-related adverse events for anti-PD-1/PD-L1 antibodies versus docetaxel

**Figure 8:**
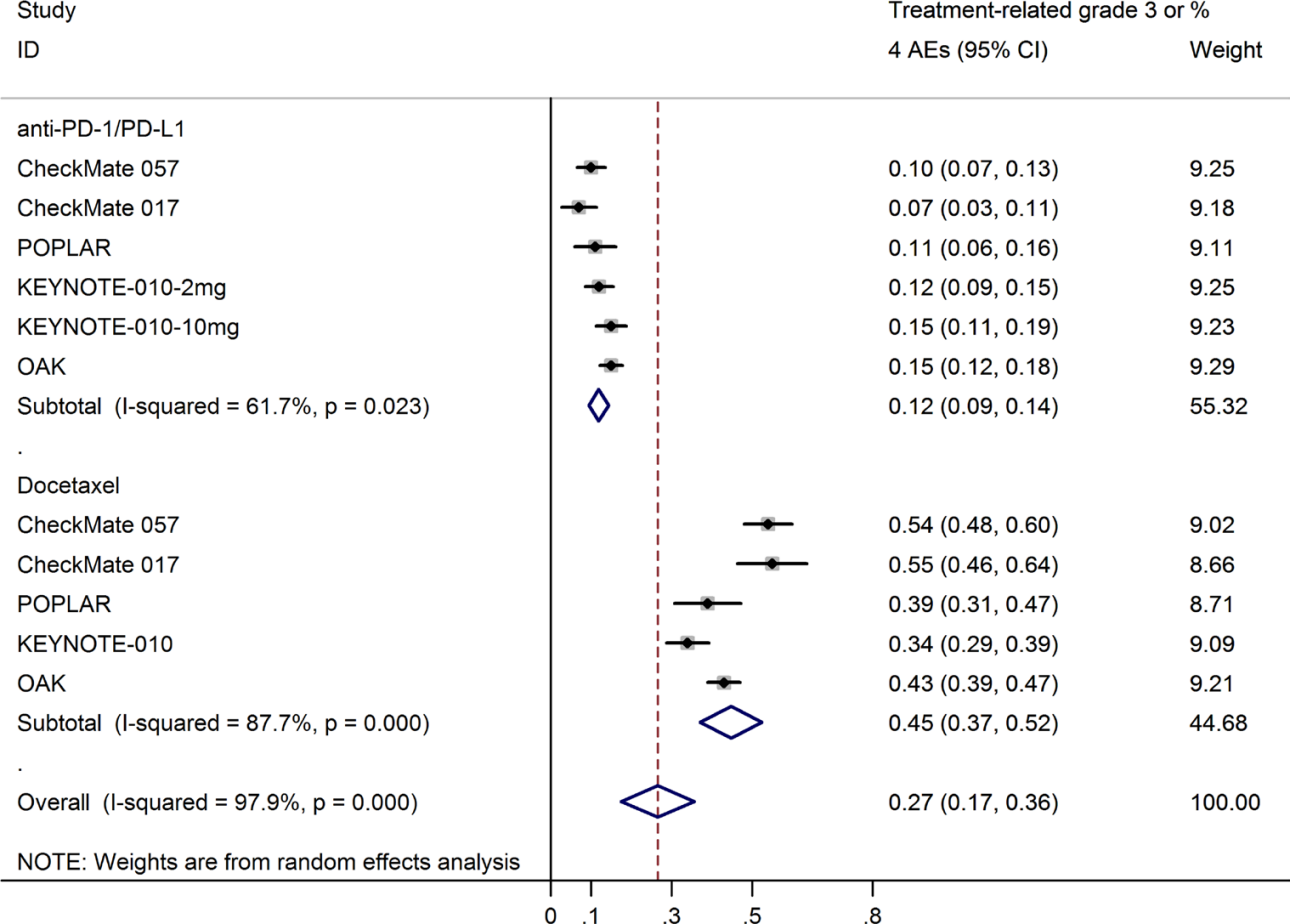
Pooled estimates of grade 3 or 4 treatment-related adverse events rates for anti-PD-1/PD-L1 antibodies and docetaxel

Supplementary Table 2 lists the grade 3 or 4 treatment-related adverse events and treatment-treated mortality in each study. As there was high heterogeneity, the grade 3 or 4 adverse events rates were calculated using a random-effects model. Meta-analysis showed that the grade 3 or 4 treatment-related adverse events rates of anti-PD-1/PD-L1 antibodies (12%; 95% CI, 9–14%) were significantly lower than that of docetaxel (45%; 95% CI, 37–52%; Figure 8), with an odds ratio of 0.18 (95% CI, 0.12–0.28; *p* = 0.000; Supplementary Figure 6). Any-grade treatment-related adverse events, including both hematologic and nonhematologic toxic events, occurred less frequently with anti-PD-1/PD-L1 antibodies than with docetaxel. The most frequently reported anti-PD-1/PD-L1 antibody–related adverse events were fatigue (15%; 95% CI, 13–16%; Supplementary Figure 7), decreased appetite (11%; 95% CI, 9–13%; Supplementary Figure 7), and nausea (10%; 95% CI, 9–11%; Supplementary Figure 7); docetaxel-treated patients most frequently had fatigue (30%; 95% CI, 25–34%; Supplementary Figure 8), alopecia (29%; 95% CI, 23–36%; Supplementary Figure 8), neutropenia (22%; 95% CI, 12–32%; Supplementary Figure 8), nausea (22%; 95% CI, 16–29%; Supplementary Figure 8), and diarrhea (20%; 95% CI, 18–23%; Supplementary Figure 8).

## DISCUSSION

Effective options are limited for patients with NSCLC whose disease progresses after first-line chemotherapy. Docetaxel was approved as a second-line treatment for advanced NSCLC based on the longer survival than that with the best supportive care, but is limited in the clinic by its relatively serious toxicity [4–5]. The tumor-expressed ligands PD-L1 and PD-L2 engage the PD-1 receptor expressed on activated T cells to induce T cell inhibition and exhaustion [14]. Many tumor cells, including NSCLC cells, overexpress PD-L1 to evade immunological surveillance [15]. Some drugs target the PD-1/PD-L1–mediated signaling pathway, including the anti-PD-1 antibodies nivolumab (BMS-936558; MDX-1106), AMP-224, pembrolizumab (MK-3475), and pidilizumab (CT-011), and the anti-PD-L1 antibodies atezolizumab (MPDL-3280A), durvalumab (MEDI-4736), and BMS-936559 (MDX-1105). In phase I/II studies, nivolumab, pembrolizumab, and atezolizumab showed durable anti-tumor activity and encouraging results on survival in previously treated advanced NSCLC [16–19], and have been approved by the US Food and Drug Administration (FDA) for treating patients with metastatic NSCLC with disease progression during or after platinum-containing chemotherapy. Based on a recent phase III randomized trial (KEYNOTE-024) [20] that compared pembrolizumab and platinum-based chemotherapy, pembrolizumab was approved by the US FDA as first-line therapy for advanced NSCLC with PD-L1 expression levels of ≥50%. A series of phase II/III studies compared the aforementioned anti-PD-1/PD-L1 antibodies with docetaxel in previously treated patients with advanced NSCLC [8–12]. Therefore, summarizing the current data is necessary for confirming the therapeutic value of anti-PD-1/PD-L1 antibodies compared with docetaxel in advanced NSCLC.

In the present meta-analysis, anti-PD-1/PD-L1 antibodies were associated with significant OS benefit over docetaxel; these results were consistent with the findings of the meta-analysis by Zhou *et al.* [21], which included CheckMate 017, CheckMate 057, and POPLAR and involved 1,141 patients. With the newly included KEYNOTE-010 study and OAK study, our meta-analysis shows that the durability of the benefit of anti-PD-1/PD-L1 antibodies is reflected in the significantly longer PFS compared to that of docetaxel. Notably, compared with patients with non-squamous NSCLC in CheckMate 057 [8], nivolumab showed better efficacy in patients with squamous NSCLC in CheckMate 017 [9], with an OR rate of more than double and a significant PFS benefit over docetaxel.

The KEYNOTE-010 study [11] enrolled patients with PD-L1 expression of TC on at least 1%, and the remaining four studies [8–10, 12] enrolled patients regardless of tumor PD-L1 expression level. The POPLAR study [10] and OAK study [12] detected PD-L1 expression level on TC as well as on IC. For uniform classification of PD-L1 expression levels, TC0 and IC0, TC1/2/3 or IC1/2/3, TC2/3 or IC2/3, and TC3 or IC3 were considered to approximate TC PD-L1 expression levels of <1%, >1%, >5%, and >50%, respectively. The subgroup analysis of PD-L1 expression levels showed an apparently greater magnitude of OS and PFS benefits of anti-PD-1/PD-L1 treatment over docetaxel in patients with higher tumor PD-L1 expression than in the overall population, while the benefit was not seen in patients with low PD-L1 expression, irrespective of the cut-off used. Consistent with the results reported by Zhou *et al.* [21], there was a trend toward longer OS and PFS as the PD-L1 expression level increased from >1% to >10%; (PD-L1 expression ≥1%: OS HR = 0.66, PFS HR = 0.81; ≥5%: OS HR = 0.55, PFS HR = 0.66; ≥10%: OS HR = 0.43, PFS HR = 0.54). Although in patients with >50% PD-L1 expression this trend was not seen in the case of data merging, we found that this trend was followed in each included trial when PD-L1 expression level increased. These results indicate a predictive association between PD-L1 expression level and sensitivity to anti-PD-1/PD-L1 treatment. Subgroup analyses of other patient characteristics revealed that specific subgroups may have driven the OS and PFS results, as suggested by the subgroup analyses of history of tobacco use and *EGFR* mutation status. We found that the advantage of anti-PD-1/PD-L1 treatment over docetaxel in OS was not shown in patients with *EGFR* mutation or in never-smokers; for PFS, docetaxel appeared superior to anti-PD-1/PD-L1 treatment in the *EGFR* mutation–positive subgroup. These results might be because never-smokers or patients with *EGFR* mutation may have low mutational heterogeneity, and tumors bearing high levels of somatic mutations have high sensitivity to immune-checkpoint inhibitors [22–23]. The negative associations between PD-L1 expression in tumors and mutated *EGFR* status in patients with NSCLC have been reported [24–25]. The relatively lower expression of PD-L1 in patients with *EGFR* mutation may affect the sensitivity to anti-PD-1/PD-L1 treatment, which is probably one reason for the findings. As the subgroups were small, the outcomes in other subgroup analyses, especially for patients outside the US/Canada, line of therapy, and *ALK* or *KRAS* gene mutation status require further verification.

The safety profile of anti-PD-1/PD-L1 treatment was more favorable in comparison with docetaxel, with lower risks of grade 3 or 4 treatment-related adverse events than docetaxel (12% vs. 45%). For any-grade adverse events, the frequencies of both hematologic and nonhematologic adverse events were substantially lower with anti-PD-1/PD-L1 antibodies than with docetaxel. Immune-related adverse events, including pneumonitis, have been reported in only a small percentage of patients treated with anti-PD-1/PD-L1 treatment, and these events were managed using established guidelines.

This study has several limitations. One is the heterogeneity of the participant inclusion criteria, where the KEYNOTE-010 study [11] enrolled patients with PD-L1 expression of TC on at least 1%, and the remaining four studies [8–10, 12] enrolled patients regardless of tumor PD-L1 expression level. There were separate nivolumab studies [8–9] for squamous and non-squamous histology, while the KEYNOTE-010, POPLAR and OAK studies enrolled patients regardless of histology [10–11]. In addition, both CheckMate studies [8–9] limited enrolment to patients who had received only one line of previous treatment for metastatic disease, whereas one-third of patients in POPLAR had received two previous lines of chemotherapy [10], and almost one-third of patients in KEYNOTE-010 had received at least two lines of previous treatment [11]. Anti-PD-L1 antibody atezolizumab, anti-PD-1 antibodies nivolumab and pembrolizumab target different molecules in the PD-1/PD-L1 pathway is another limitation, although similar outcomes for both antibody drugs have been demonstrated in clinical studies. Finally, as the subgroups were small, future studies should verify the results for OS and PFS in subgroup analyses after expanding the sample size.

In conclusion, the present meta-analysis demonstrates that anti-PD-1/PD-L1 treatment led to superior survival benefit with an improved safety profile over that for docetaxel in patients with previously treated advanced NSCLC. Patients with positive PD-L1 expression may benefit more from anti-PD-1/PD-L1 therapy. The identification of relevant biomarkers with sufficient sensitivity and specificity for predicting which patients are most likely to benefit from this therapy is needed in future studies.

## MATERIALS AND METHODS

### Data sources

This meta-analysis was conducted according to the PRISMA guidelines. We systematically searched the PubMed, EMBASE and Cochrane library databases for eligible studies. The search time was from database inception to April 1, 2017. The combination of free-text words and MeSH terms were used as follows: (lung neoplasms/carcinoma/non-small-cell lung cancer/NSCLC/lung cancer) AND (programmed cell death 1 receptor/PD-1/programmed death-ligand 1/PD-L1) AND (nivolumab/MDX-1106/ONO-4538/BMS-936558/opdivo/atezolizumab/MPDL3280A/tecentriq/RG7446/pembrolizumab/lambrolizumab/keytruda/MK-3475) AND (docetaxel/docetaxol). Reference lists from eligible studies were also searched thoroughly for potential relevant studies.

### Study selection, meta-analysis inclusion criteria, and data extraction

The publications identified were carefully screened. Only the most recent randomized clinical trials were included in the meta-analysis. Preclinical studies, case reports, and reviews were excluded. Two reviewers (Qi Jiang, Mixue Xie) screened all publications identified based on our inclusion criteria. In the event of disagreement between the two reviewers, we obtained and inspected the full-text article independently. In total, five studies were included in the final analysis.

The inclusion criteria were: (1) randomized controlled trial; (2) patients with advanced or metastatic NSCLC after failure of previous treatments; (3) anti-PD-1/PD-L1 antibodies treatment as compared with chemotherapy; (4) published in English; (5) reported OR rate, toxicity data, or at least one form of survival data. The extracted data included: (1) study characteristics (author, publication time, research period, study type, prior therapy); (2) patient characteristics (age, sex, histology, tobacco use history); (3) PD-1/PD-L1 therapy and docetaxel regimen; and (4) outcome measures (CR rate, PR rate, OR rate, number of patients with stable disease [SD]; number of patients with progression of disease [PD]; HRs for OS and PFS, grade 3–5 treatment-related adverse events rates).

### Quality assessment and statistical analysis

The methodological quality of trials was assessed by the Cochrane risk of bias tool. All the meta-analyses were performed using Review Manager 5.3 (Cochrane Collaboration, Oxford, UK). Pooled estimates of OR rate and grade 3 or 4 treatment-related adverse events rate were computed when there was sufficient reporting of these measures. The odds ratio was used for comparing the OR rates and adverse events rates. Time-to-event outcomes (OS or PFS) were analyzed as HRs and pooled according to effect size and 95% CIs. We assessed heterogeneity in the results of the trials using the χ^2^ test of heterogeneity and the I^2^ measure of inconsistency. We considered heterogeneity present when the *p*-value of the Cochran Q test was <0.05 and the I^2^ statistic was >50%. We performed meta-regression or subgroup analyses to find the source of heterogeneity [26, 27]. If it was necessary, sensitivity analysis was also performed. The random-effects model was used for meta-analysis if there was significant heterogeneity. Publication bias was evaluated by Deek’s funnel plot visually and analytic methods (Begg or Egger test) [28, 29]. A statistical test with *p* < 0.05 was considered significant. All statistical analyses were performed using the meta-analysis command in STATA (version 12.0 for Windows; Stata Corp LP, College Station, TX).

## SUPPLEMENTARY MATERIALS FIGURES AND TABLES



## Abbreviations

PD-1: programmed death receptor 1
PD-L1: programmed death-ligand 1
NSCLC: non-small cell lung cancer
CI: confidence interval
OS: overall survival
HR: hazard ratio
PFS: progression free survival
EGFR: epidermal growth factor receptor
ECOG: eastern cooperative oncology group
RECIST: response evaluation criteria in solid tumors
KRAS: kirsten rat sarcoma
ALK: anaplasticlymphoma kinase
OR: objective response
CR: complete response
PR: partial response
TC: tumor cells
IC: tumor-infiltrating immune cells
CNS: central nervous system
PD-L2: programmed death-ligand 2
FDA: the US Food and Drug Administration
SD: stable disease
PD: progression of disease
