# Supplementary material for: Anti-PD-1/PD-L1 antibodies versus docetaxel in patients with previously treated non-small-cell lung cancer
Source: Oncotarget. 2017 Dec 21;9(7):7672–83. doi: 10.18632/oncotarget.23584 (PMC5800935; doi:10.18632/oncotarget.23584)
Supplement: Supplementary file 1 [file oncotarget-09-7672-s001.pdf]

Anti-PD-1/PD-L1 antibodies versus docetaxel in patients with previously treated non-small-cell lung cancer

SUPPLEMENTARY MATERIALS

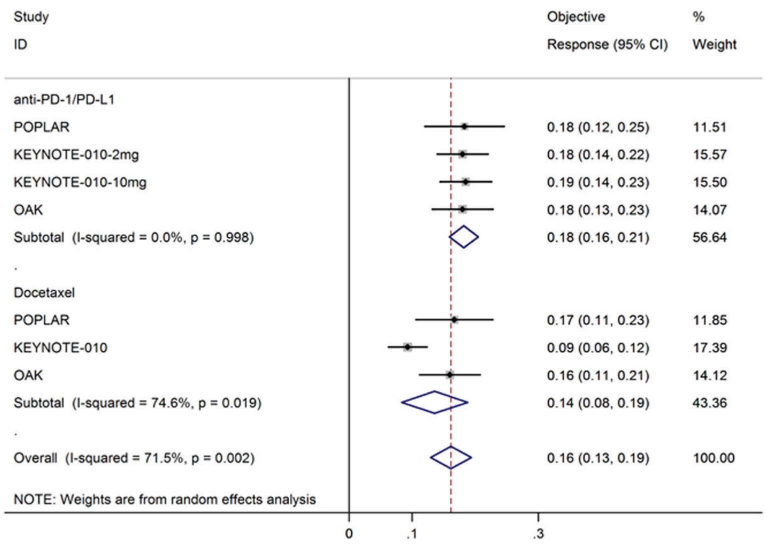

Supplementary Figure 1: Pooled estimates of OR rates for anti–PD-1/PD-L1 antibodies and docetaxel in patients with PD-L1 expression ≥1%.

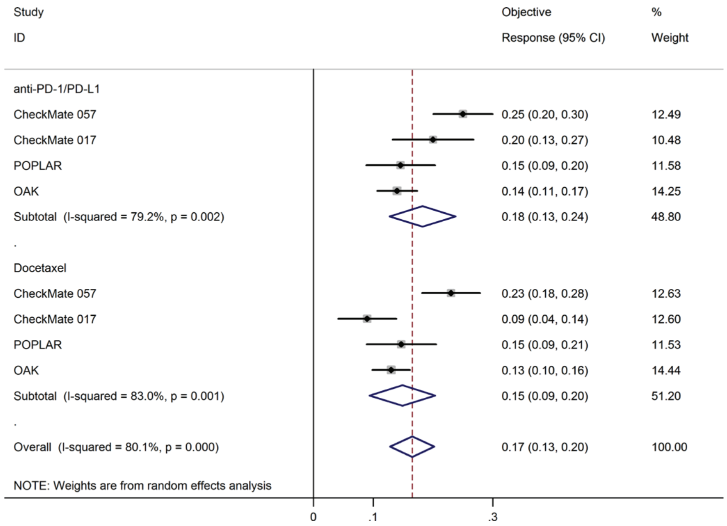

Supplementary Figure 2: Pooled estimates of OR rates for anti–PD-1/PD-L1 antibodies and docetaxel in patients with no limited PD-L1 expression.

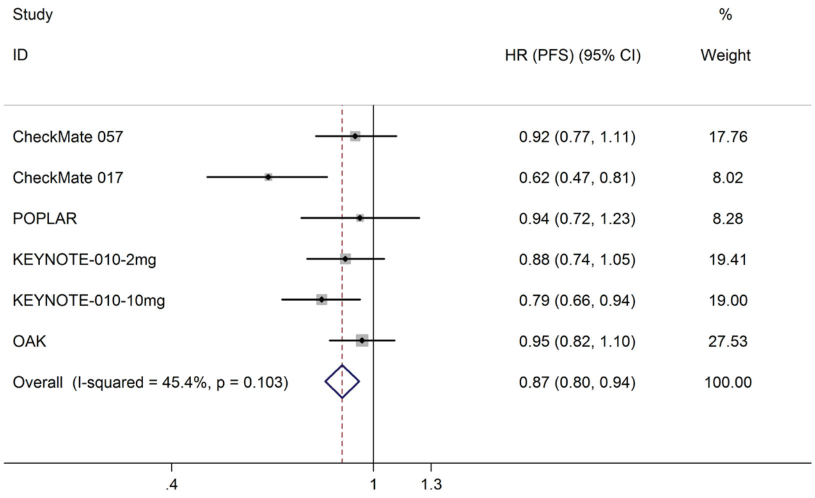

Supplementary Figure 3: Meta-analysis of PFS.

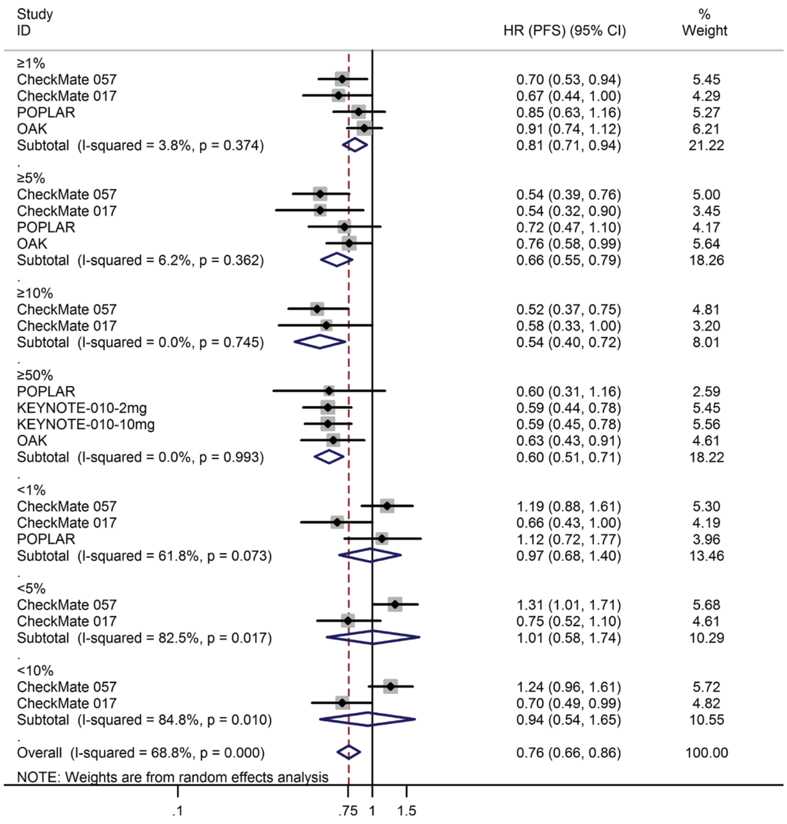

Supplementary Figure 4: Meta-analysis of PFS in patients with 1%, 5%, 10%, and 50% PD-L1 expression.

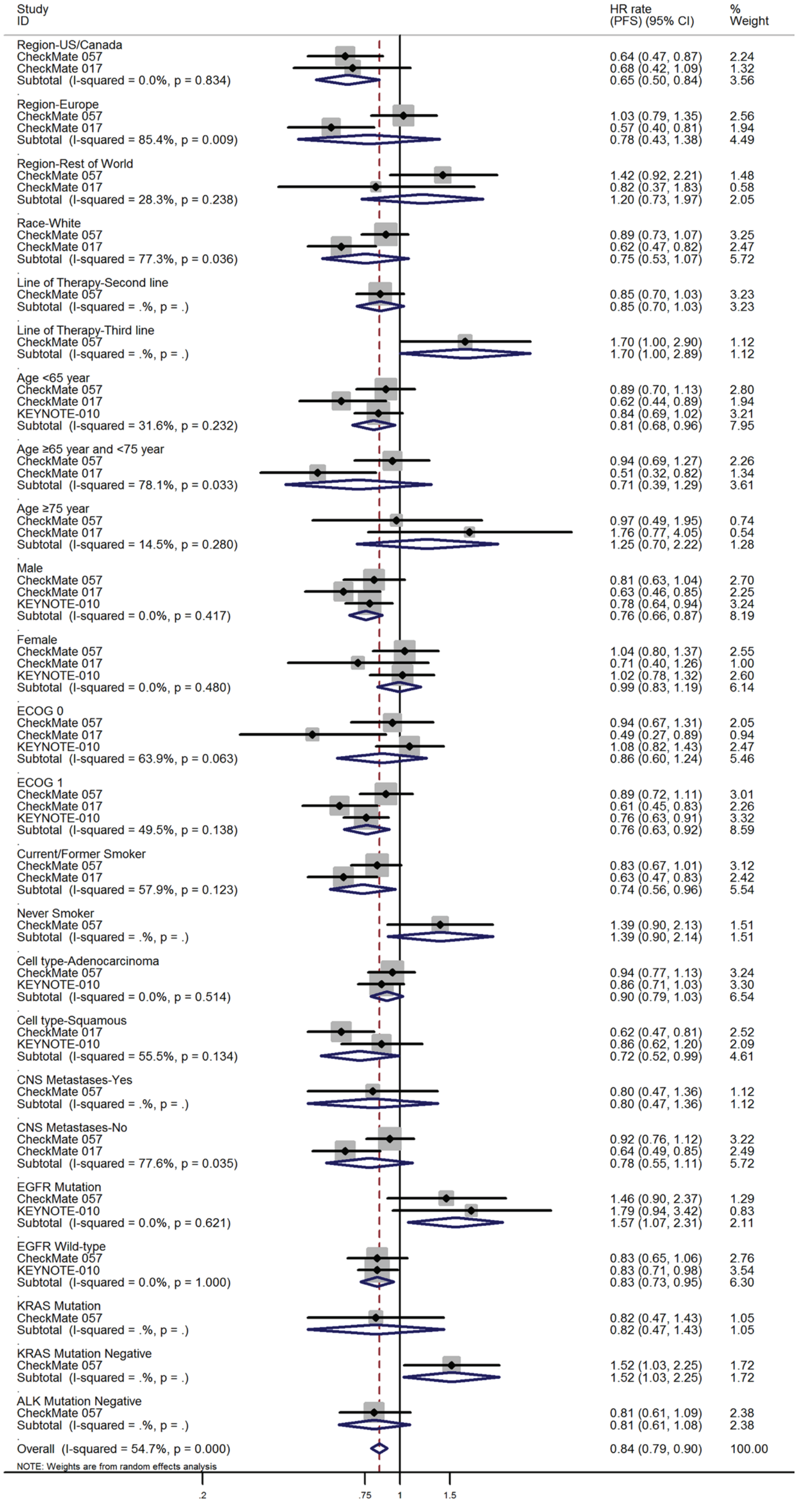

Supplementary Figure 5: Subgroup analyses of the associations between PFS and patient features.

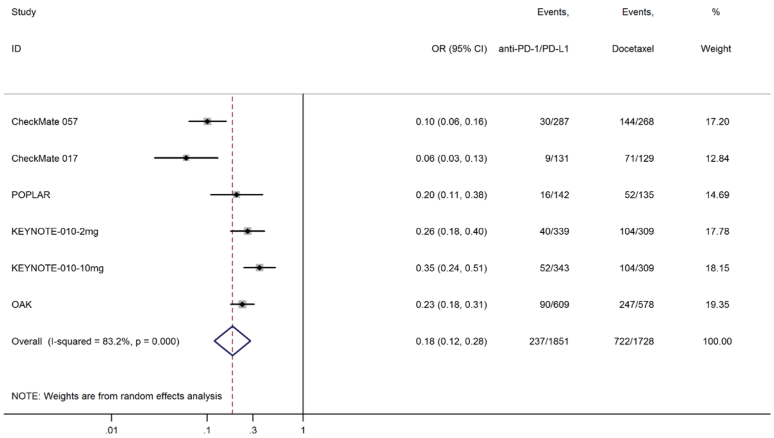

Supplementary Figure 6: Meta-analysis of grade 3 or 4 drug-related adverse events rates.

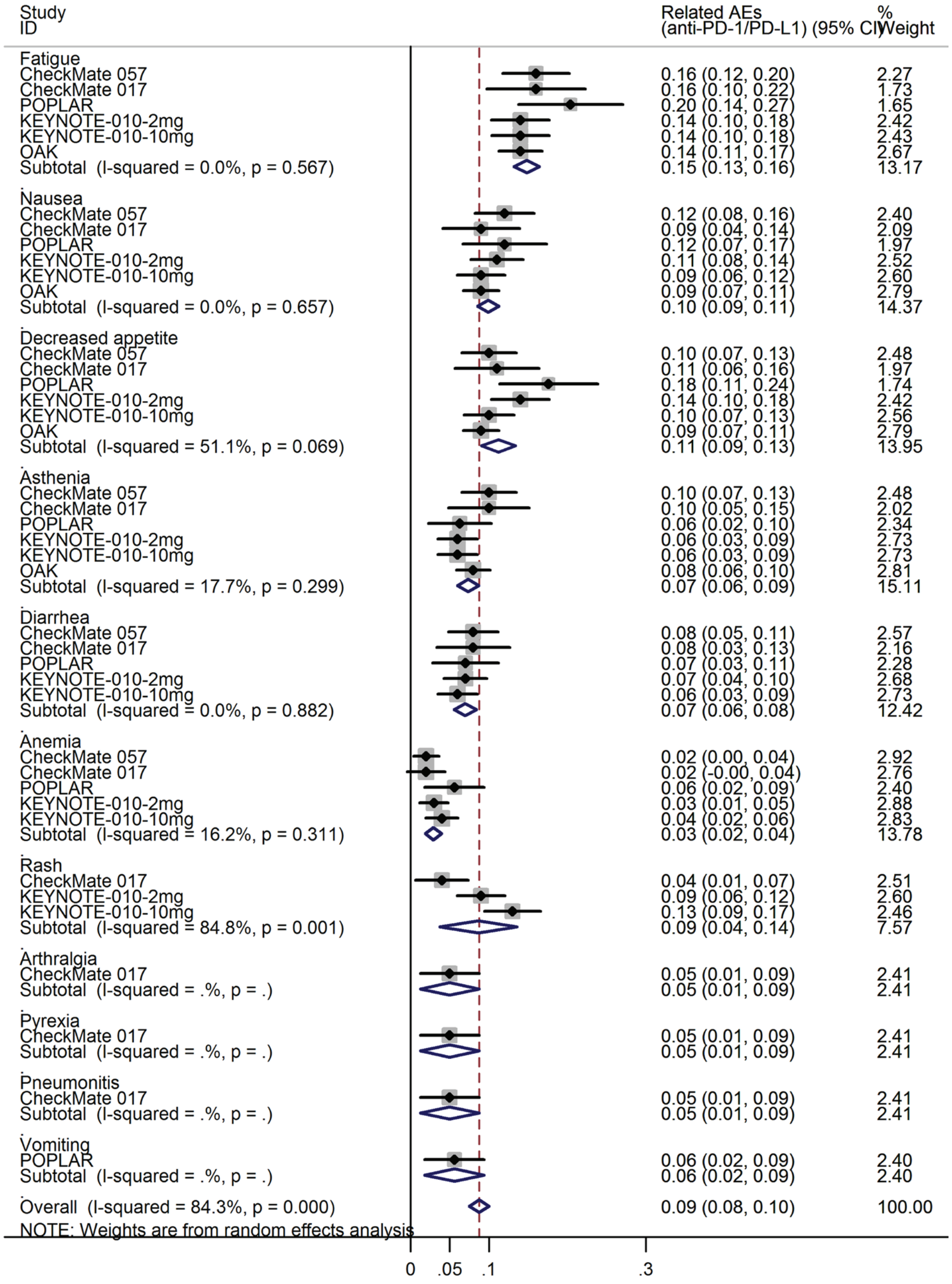

Supplementary Figure 7: Pooled estimates of any-grade treatment-related adverse events rates for anti–PD-1/PD-L1 antibodies.

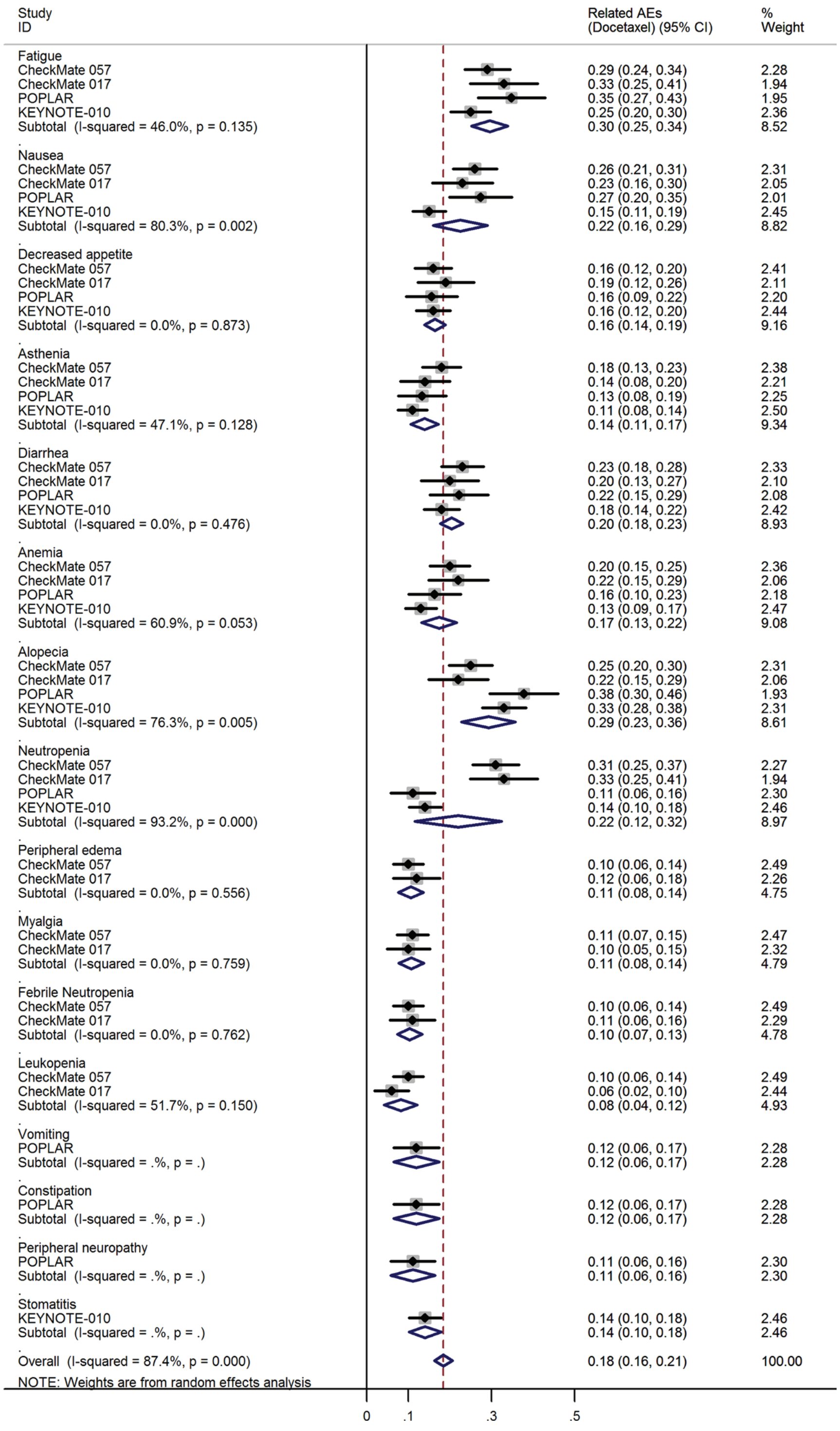

Supplementary Figure 8: Pooled estimates of any-grade treatment-related adverse events rates for docetaxel.

Supplementary Table 1: Univariate meta-regression analysis for the potential variables between studies

Covariates	Coefficient	Standard error	*t*	*p* value	95% Confidence interval	
Sample size	−0.000148	0.0009	−0.16	0.880	−0.0027	0.00241
Race	0.491	0.507	0.97	0.388	−0.916	1.897
Duration-Docetaxel	−0.467	0.441	−1.06	0.349	−1.69	0.758
Duration-anti PD-1/PD-L1	0.688	0.550	1.25	0.279	−0.839	2.215
PD-L1 expression	0.611	0.223	2.73	0.052	−0.997	1.231

Supplementary Table 2: Grade 3–5 treatment-related adverse events

Author and study	Drug	Treatment-related Grade 3 or 4 adverse events no. (%)	Treatment-related Grade 5 adverse events (Deaths) no. (%)
Borghaei [8] CheckMate 057	Nivolumab (PD-1 antibody)	30 (10)	1 (0.4)
	Docetaxel	144 (54)	1 (0.4)
Brahmer [9] CheckMate 017	Nivolumab (PD-1 antibody)	9 (7)	0
	Docetaxel	71 (55)	3 (2.3)
Fehrenbacher [10] POPLAR	Atezolizumab (PD-L1 antibody)	16 (11)	1 (0.7)
	Docetaxel	52 (39)	3 (2.2)
Herbst [11] KEYNOTE-010	Pembrolizumab (2 mg/kg) (PD-1 antibody)	40 (12)	3 (0.9)
	Pembrolizumab (10 mg/kg) (PD-1 antibody)	52 (15)	3 (0.9)
	Docetaxel	104 (34)	5 (1.6)
Rittmeyer [12] OAK	Atezolizumab (PD-L1 antibody)	90 (15)	0
	Docetaxel	247 (43)	1 (0.2)
